# Spontaneous memory strategies in a videogame simulating everyday memory tasks

**DOI:** 10.1177/17470218231183958

**Published:** 2023-07-03

**Authors:** Matti Laine, Jussi Jylkkä, Liisa Ritakallio, Tilda Eräste, Suvi Kangas, Alexandra Hering, Sascha Zuber, Matthias Kliegel, Daniel Fellman, Juha Salmi

**Affiliations:** 1 Department of Psychology, Åbo Akademi University, Turku, Finland; 2 Department of Psychology, University of Helsinki, Helsinki, Finland; 3 Department of Developmental Psychology, Tilburg University, Tilburg, The Netherlands; 4 Faculty of Psychology and Educational Sciences, University of Geneva, Geneva, Switzerland; 5 Centre for the Interdisciplinary Study of Gerontology and Vulnerability, University of Geneva, Geneva, Switzerland; 6 Swiss National Centre of Competence in Research LIVES—Overcoming Vulnerability: Life Course Perspectives, Lausanne & Geneva, Switzerland; 7 Department of Neuroscience and Biomedical Engineering, Aalto University, Espoo, Finland

**Keywords:** Prospective memory, strategies, executive functions, mnemonics, everyday behaviour, videogaming, serious gaming

## Abstract

People can use different internal strategies to manage their daily tasks, but systematic research on these strategies and their significance for actual performance is still quite sparse. Here we examined self-reported internal strategy use with a 10-block version of the videogame EPELI (Executive Performance in Everyday LIving) in a group of 202 neurotypical adults of 18–50 years of age. In the game, participants perform lists of everyday tasks from memory while navigating in a virtual apartment. Open-ended strategy reports were collected after each EPELI task block, and for comparison also after an EPELI Instruction Recall task and a Word List Learning task assessing episodic memory. On average, 45% of the participants reported using some strategy in EPELI, the most common types being grouping (e.g., performing the tasks room by room), utilising a familiar action schema, and condensing information (e.g., memorising only keywords). Our pre-registered hypothesis on the beneficial effect of self-initiated strategy use gained support, as strategy users showed better performance on EPELI as compared with no strategy users. One of the strategies, grouping, was identified as a clearly effective strategy type. Block-by-block transitions suggested gradual stabilisation of strategy use over the 10 EPELI blocks. The proneness to use strategies showed a weak but reliable association between EPELI and Word List Learning. Overall, the present results highlight the importance of internal strategy use for understanding individual differences in memory performance, as well as the potential benefit for internal strategy employment when faced with everyday memory tasks.

## Introduction

The use of different mnemonic strategies has a very long history (Yates, 1966) and extensive research evidence indicates that strategy employment can boost one’s memory performance (for an overview, see, for example, Bellezza, 1981). The study of memory strategies is thus important for understanding the cognitive processes involved in task performance and the sources of inter- and intra-individual differences in task outcomes. Task features can have strong effects on spontaneous strategy use (e.g., Morrison et al., 2016), calling for the study of strategic behaviour in different task paradigms. Here, we examined adults’ spontaneous strategy use and its relationship with objective task performance in a new videogame designed to tap goal-directed behaviour and everyday memory in life-like everyday contexts (Jylkkä et al., 2023; Seesjärvi et al., 2022). The present study was expected to shed light on adults’ self-generated memory strategies in life-like situations and their significance for actual memory performance, an issue that has not received much systematic research.

Our focus was on internal, self-generated memory strategies in a task where the use of external memory aids was prohibited. The definition we used is as follows: internal memory strategies are conscious, verbalisable ways to solve a memory task. Previous studies on internal strategy employment in episodic or working memory have repeatedly observed enhanced performance in conjunction with strategies, albeit the degree and duration of these effects has varied. This is true both for studies examining spontaneous strategy use (Dunlosky & Kane, 2007; Dunning & Holmes, 2014; Fellman et al., 2020; Forsberg et al., 2020; Laine et al., 2018; Malinovitch et al., 2021; McNamara & Scott, 2001; Turley-Ames & Whitfield, 2003; Waris, Fellman, et al., 2021; Waris, Jylkkä, et al. 2021) and for studies that sought causal evidence for the role of strategies in memory performance through manipulation of participants’ strategic choice (e.g., Bailey et al., 2014; Borella et al., 2017; Carretti et al., 2007; Fellman et al., 2020; Forsberg et al., 2020; Laine et al., 2018; Malinovitch et al., 2021; McNamara & Scott, 2001; Turley-Ames & Whitfield, 2003).

The present study is relevant to prospective memory (PM) research, albeit our task differs from classical experimental PM tasks, as we discuss below. Thus, we dwell briefly on previous literature that has highlighted the significance of spontaneous strategies in PM performance. Overall, metacognitive processes, responsible for strategy generation, task initiation, and monitoring (Chein & Schneider, 2012), are thought to guide the way a PM task is tackled (e.g., Rummel & Meiser, 2013). Although some strategic aspects of PM behaviour are observable either indirectly (resource allocation to an ongoing non-PM task) or directly (monitoring through clock-checking in time-based PM tasks), many internal strategies are accessible only through self-reports. As internal strategies are the topic of our study, we will briefly review previous research that has specifically addressed self-generated and self-reported PM internal strategy use. This rather limited literature has addressed strategy use in imaginary everyday PM situations, daily life, and in laboratory PM tasks.

Imaginary everyday memory scenarios are potentially relevant for understanding real-life strategy use, albeit the actual employment of strategies is not measured. Intons-Peterson and Fournier (1986) examined university undergraduates’ spontaneous internal and external strategy choices in different imaginary situations. Both the imaginary situations (e.g., “You are going to begin a two-day trip tomorrow to visit a place you have never seen before. You look at a map for directions. What would you do to try to remember where to go if you couldn’t take the map with you?”) and possible strategy types were provided to the participants. For PM situations, internal and external strategy use was proposed by 23.9% and 25.2% of the participants, respectively. The participants’ strategy choices were also influenced by the PM task at hand (verbal vs spatial). Across all the past and future imaginary situations, the most chosen internal strategies were mental rehearsing (13.0%) and mental retracing (thinking about something that happened before, or that may happen, step by step, in an attempt to remember something; 11.9%). A more recent related study by Penningroth and Scott (2013) employed an imaginary PM task to elicit open-ended strategy reports from their undergraduate participants. While external strategies were clearly most frequent (the use of note, timer, or calendar appeared in 12%–53% of the reports), one internal strategy, mental rehearsal, was listed by circa 3%–6% of their participants.

Another, perhaps more realistic approach to everyday PM strategy use has been the use of questionnaires that probe participants’ strategy employment in highly relevant PM tasks such as taking one’s medication. For example, Gould et al. (1997) examined medication adherence in older adults and identified several common ways participants used in remembering to take one’s medicine. These included associating pill-taking with certain regular daily activities, mentally repeating the instructions, and concentrating hard on the information related to medicine intake. Another survey on medication adherence by Blaskewicz Boron et al. (2013) indicated that 43% of their elderly responders employed either external or internal strategies for remembering to take their medication. Two of the seven strategy alternatives that the participants had to choose from could be considered as internal, namely, association (using an activity or event to help in remembering to take medication) and planning (thinking ahead about when one will take medication). Of the strategy users, 40% and 23% reported employing association and planning, respectively.

The third approach to examine internal PM strategy use has been the study of strategies in conjunction with PM experiments. In a recent study, Reese-Melancon et al. (2019) examined self-reported strategies in event-based laboratory PM tasks. Their study indicated that half of the participants reported strategy employment. By far, the most common strategies were monitoring/maintenance rehearsal (intentionally bringing the PM task to mind throughout the ongoing task) and consciously keeping up a sensory-motor readiness to perform the PM part of the task (e.g., tapping the finger over the PM-related response button). Reese-Melancon et al. (op.cit.) did also address the important link between strategy use and actual PM performance, finding that self-reported strategy use was associated with a significantly better performance on the more demanding task condition (a non-focal event-based PM task).

The three lines of research reviewed above suggest that part of adults use self-generated internal memory strategies to remember future actions, but their type and frequency show considerable variation that prompts further research. Moreover, the relationships between internal strategy use and objectively measured PM performance have received only limited attention (see Reese-Melancon et al., 2019).

In contrast to the rather few and varied studies on self-generated strategy use in PM, several intervention studies have examined the effects of PM strategy training on task performance. A recent meta-analysis by Jones et al. (2021) demonstrated the efficacy of such training regimes. The most frequently taught strategy has been implementation intention (creating an environmental cue for an intention and speaking it aloud: “If I see cue X, I’ll perform Y”). Other PM strategy training regimes have included a visual imagery strategy (imagining oneself performing Y when seeing cue X), spaced retrieval (recall of information over progressively longer periods of time), increased self-reflection (“Am I forgetting anything?”), or a combination of multiple strategies. Although these intervention studies have consistently documented the facilitative effects of strategy use on PM performance and thus provided important causal evidence for the role of strategies in PM performance, they do not address spontaneous strategy use that is relevant for understanding individual differences in PM performances. Moreover, Jones et al. (2021) noted that the ecological validity of the PM assessments employed has been low.^1^ Thus, to clarify PM strategy use in more life-like contexts, further research with new paradigms is called for.

EPELI (Executive Performance in Everyday LIving) is a recently developed videogaming task where participants move around in a virtual apartment, performing a list of common household tasks (e.g., preparing food, cleaning the place, getting ready to go to a hobby) orally given by an avatar. It provides a rich yet controllable context for quantitative measurement of goal-directed behaviour and everyday memory in a more life-like setting by mirroring everyday chores that need to be planned and executed around the house. It should be pointed out that EPELI differs from the many laboratory PM paradigms that include a previously instructed PM task embedded within an ongoing task (Einstein & McDaniel, 1990). Instead, in the current EPELI version, the participant is confronted with multiple to-be-remembered tasks that are performed immediately and that are not embedded with an ongoing task. Thus, EPELI belongs to the family of complex action memory tasks that have been designed to highlight especially the planning/executive component of memory performance (e.g., the Six Element test and the Multiple Errands test by Shallice & Burgess, 1991; the modified Six Element PM test by Kliegel et al., 2000; the Breakfast task by Craik & Bialystok, 2006) where strategy use becomes a central issue.

The first study with EPELI, conducted with school-age children, found that it differentiated children with attention deficit hyperactivity disorder (ADHD) diagnosis from neurotypical participants, and that efficacy on EPELI game play correlated with parent-rated executive problems and ADHD symptoms (Seesjärvi et al., 2022). Compared with classical laboratory paradigms, the more naturalistic situations and the freedom to choose one’s actions in EPELI provide an innovative and ecologically more relevant way to examine spontaneous memory strategy use and its relationships with actual task performance. This inquiry is also relevant in terms of potential interventions: recognising and facilitating self-generated strategy use can provide a particularly effective avenue for cognitive training (e.g., Scarff et al., 2022). In the present online study, we employed a 10-block flat screen adult version of EPELI and collected open-ended strategy reports from the participants after each block.

The present pre-registered study (https://osf.io/m7c9a; “Study 2”) set one a priori hypothesis and two research questions. Based on the extensive evidence for the facilitative role of strategies in episodic and working memory performance (see above), our hypothesis was that EPELI performance shows a positive association with spontaneous strategy use. The first research question addressed the possible evolvement of strategy use over the 10 EPELI task blocks. Earlier research has indicated a rapid stabilisation of the use of mnemonic strategies (Waris, Fellman, et al., 2021; Waris, Jylkkä, et al. 2021), but that evidence comes from experimental working memory and episodic memory tasks. A similar evolvement in EPELI would speak for its generality, as one would expect within the cognitive skill learning framework (Chein & Schneider, 2012). The second research question concerned the congruity of participants’ strategy use versus non-use in EPELI and two other memory tasks (EPELI Instruction Recall task, Word List Learning task). Strategy use appears to vary widely even within a memory domain (Morrison et al., 2016), and it is not clear to what extent individuals could be characterised by strategy proneness, that is, the degree to which they tend to resort to strategies when faced with different memory tasks. Elucidation of commonly used mnemonic strategies and their evolvement within the EPELI task may increase our understanding of the underlying mechanisms of complex everyday memory performance. Ultimately, this has the potential to inform the development of personalised strategy instructions that could be used in real life by people suffering from memory problems.

## Methods

### Participants

Research ethics screening was conducted by the Ethics Board of the Departments of Psychology and Logopedics at the Åbo Akademi University, Turku, Finland. Anonymous participants were recruited on the crowdsourcing site Prolific (prolific.co), targeting both neurotypical participants and adults with diagnosed ADHD (both groups of age 18–50, currently living in the United Kingdom, first language English). They received monetary compensation for their participation. The present study focusses only on part of the sample and the performance data, namely, healthy participants and their strategy use in EPELI. The rest of the data, including both questionnaires and other cognitive tasks (see Jylkkä et al., 2023), will be reported in separate papers.

The data collection in August–December 2021 proceeded in three stages, two prescreens and the study proper that encompassed five sessions on five separate days (see Jylkkä et al., 2023, for further details). The pre-registered goal was to gather data from at least 250 neurotypical individuals and 100 people with ADHD, but we had to run the prescreens with much larger groups to obtain a sufficient number of adults with ADHD that suited to the inclusion/exclusion criteria. Thus, we had 14,443 participants in the very short first prescreen that included a question on possible ADHD/attention deficit disorder (ADD) diagnosis and part A of the Adult ADHD Self-Report Scale (Adler et al., 2006).

At the next step, 1,513 non-ADHD participants on a first-come-first-serve basis took the second circa 10-min prescreening that consisted of several health-related questionnaires. To pass the second prescreening, the following criteria had to be met: normal or corrected-to-normal vision, no colour blindness; no neurodevelopmental disorders; no neurological illness that affects the participant’s current life; never diagnosed with severe depression, bipolar disorder, psychosis, or schizophrenia across the lifespan; and no self-reported substance-abuse problem. Moreover, the following eligibility criteria based on the *Diagnostic and Statistical Manual of Mental Disorders*, fifth edition (*DSM*-5) Self-Rated Level 1 Cross-Cutting Symptom Measure—Adult (Bravo et al., 2018) were applied: no reported suicidality (i.e., Score 0 in Item 11), and sum scores of less than 3 in the depression, mania, and anxiety domains (i.e., at most “mild” symptoms, or a response indicating occurrence of the symptom not more than during “several days” over the last 2 weeks).

Again based on the first-come-first-serve principle, 293 healthy individuals who had fulfilled the criteria set in Prescreening 2 took part in the experiment proper. In this group, the following exclusions were made: participant reported cheating or intoxication in any of the five sessions (*n* = 32), missing background data that were necessary to determine eligibility in the study (*n* = 6), lost EPELI data due to technical problems (*n* = 11), missing EPELI data at least in one EPELI block (*n* = 3), and missing strategy report at least in one EPELI block (*n* = 39). We performed listwise exclusions to simplify the analysis and the interpretation of the results. No univariate outliers (scoring three times the interquartile range above or below the first or the third quartile in a given dependent variable) were found. Thus, the final participant group subjected to the statistical analyses included 202 individuals. Their mean age was 32 years, and 71% of the participants were females.

### Test sessions

The experiment proper encompassed five sessions. EPELI was always administered in the first session and the diary questions (see below) were administered in every session. Other cognitive tasks not detailed here (two classical PM tasks, Continuous Performance task, Fluid Intelligence task, Instruction Recall task, Word List Learning task; only correlations with strategy use in the latter two tasks are reported here) were counterbalanced between the participants, randomly allocating the participants into one of the four counterbalanced task orders. The five testing sessions were performed on separate weekdays with at least a 12-hr interval in-between, and all sessions were to be completed within 14 days. Each session took approximately 40 min, and the complete duration of testing was circa 3 hr and 20 min. Only those participants who completed EPELI in their first session were allowed to partake in the other sessions.

### The EPELI game

**Figure 1.**
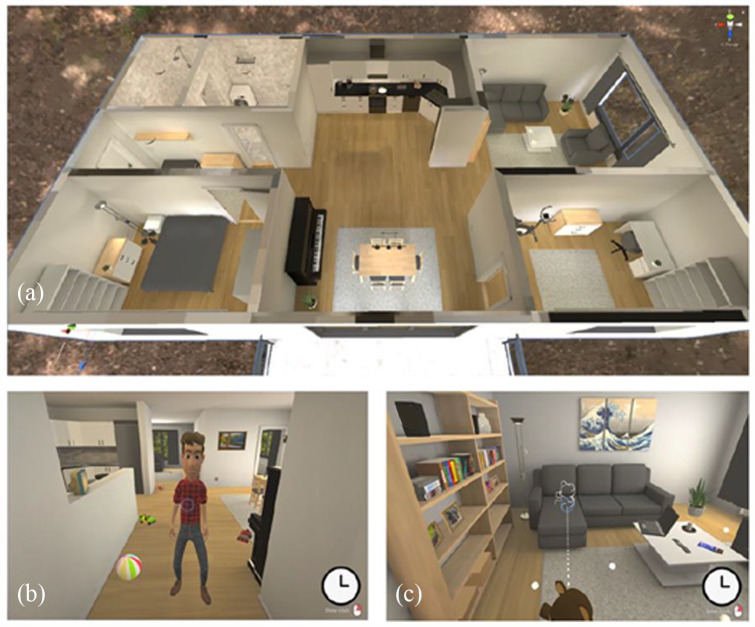
The EPELI game. The participant is to perform everyday chores from memory in a virtual apartment. The floorplan (a) is not available to the participant. The list of tasks for each block is given orally by the virtual character Vincent (b). In this game version, the event-based memory task is to place the teddy bear, when present among the toys scattered on the floor, on the sofa (c). The participant moves around by clicking on white hotspots on the floor. A clock with running time can be opened by clicking the right mouse button.

The EPELI game started with guided hearing threshold detection that adjusted the sound volume levels to equal level across all participants, after which the participants found themselves in the lobby of a virtual apartment. A virtual character named Vincent, appeared in front of the participants, welcoming them and giving instructions to the game. This was followed by a practice session where participants had to move an apple from one room to another. Visual field of view in the game was controlled by mouse and a crosshair was visible at the centre of the screen. The participants moved around the apartment by clicking on white circles on the floor, and they were able to move directly to any visible circle. A clock with running time (reset at the beginning of each block) was available in the lower right corner by clicking the right mouse button. Objects could be grabbed and laid on surfaces by clicking on them. Several of the objects were interactable, for example, closets that could be opened, toys scattered on the floor that could be manipulated, or the piano in the living room that one could play. See Figure 1 for still images of the EPELI game.

The present adult version of EPELI contained 10 blocks with eight tasks per block. In the beginning of each block, Vincent orally introduced a theme (e.g., “You have invited your friend to dinner. You are sitting on your bed. Listen carefully to the things you should do in the apartment. They are as follows.”) and gave a list of seven to eight everyday tasks. Three types of memory tasks appeared in Vincent’s block-wise instructions. Most of the tasks (altogether 70) were labelled as *free tasks* that did not have any specified trigger event or time point (seven per block; e.g., “Pick up the salad from the fridge and bring it to the dining table,” “Switch on the table lamp on the work desk”). Five tasks were *time-based memory tasks* (to be performed at a given time point, e.g., “After one minute, warm up briefly with the exercise bike in the study”). These appeared in five blocks, (i.e., one per block), but never together with the event-based task that is described next. The *event-based memory task* was ongoing throughout the EPELI session, to be performed whenever a cue was present. The cue was a teddy bear, which was to be put on the sofa. The teddy was present in five of the 10 blocks (the ones that did not include a time-based memory task), located among toys scattered on the floor (see Figure 1c). The instruction to this event-based task was given by Vincent only once before the first block started. Hence, the task lists for the blocks did not include the event-based instruction anymore. Each block thus contained seven free tasks, and additionally, either one time-based task or one event-based task, making in total eight tasks in each block. Sixty of the free tasks were in line with the general schema of the task block that was provided by Vincent, while 10 of the free tasks (one per block) were non-schema-based, that is, unrelated to the schema in question (e.g., during preparation of a vegetable soup, one had to run a bath). This was done to reduce predictability of the task lists.

In the present study, we employed three previously defined EPELI outcome variables, namely, total score, task efficacy, and navigation efficacy (see Seesjärvi et al., 2022). For each block, total score depicts the number of correctly performed subtasks, task efficacy corresponds to the proportion of relevant actions out of all actions (excluding clicks on the waypoints that enable moving around in the environment; “relevant” means actions that contribute to completing the given tasks), and navigation efficacy is the total score divided by covered distance. These three variables were chosen here as they reflect overall performance on a block and because higher scores on these three variables unequivocally reflect better performance, enabling us to test the pre-registered hypothesis that strategy use is associated with better success in EPELI.

After each block, the participants provided a written response to the following open question: “Please describe in as much detail as possible how you solved the previous set of tasks.” These responses were used to code individual block-wise strategy use in EPELI, as described below. For studying the associations of strategy use between EPELI and the other two memory tasks (Word List Learning and EPELI Instruction Recall, described below), we also coded for each of the three tasks the number of blocks where a participant reported strategy use (0–10 for the 10-block EPELI, 0–3 for the 3-block Word List Learning, 0–1 for the single-block EPELI Instruction Recall).

### Word List Learning task

This episodic memory/learning task was adopted from the work of Waris, Fellman, et al. (2021). It consisted of a list of 18 common nouns that was presented three times in randomised order. After each presentation, the participants were to recall as many words as possible. Each word was shown on the screen for 1 s, separated by a 1-s blank-screen interval. After the final word in a list had been shown, a multiple-choice distractor task appeared. The distractors were arithmetical tasks (e.g., 9 + 8 – 7 + 6 = ?) to wash out the words from working memory.

After the distractor task, the participants were to type in the words in any order in a set of 18 boxes. Words had to be typed correctly, but non-letter characters or spaces were permitted before or after the word. The dependent measure was the number of correctly recalled words per list.

After each of the three word list presentations, the participants gave a written response to the following question: “Please describe in as much detail as possible how you solved the previous word list task (not the math task). That is, how did you try to memorize the words?.” The responses were used for coding their strategies (see section “Strategy coding for the memory tasks”).

### EPELI Instruction Recall task

In this task, the participants received a list of eight instructions (e.g., “Take off your overcoat and hang it in the coat rack”) similar to the ones they had to memorise in the EPELI blocks. The instructions were presented one at a time on the computer screen, for the duration of 4 s each. The theme of the list was “Returning home from a grocery store.” Immediately after being presented with the list, the participants were to write down as many instructions as they remembered. All elements in each instruction (e.g., “take off your overcoat” and “put it in the coatrack”) had to be reported correctly to receive points. The dependent variable was the total number of correctly recalled instructions (0–8). Right after the Instruction Recall task, the participant responded to the following open question: “Please describe in as much detail as possible how you solved the task. That is, how did you try to memorize the instructions?” The responses were used for coding the participants’ strategies on this task (see section “Strategy coding for the memory tasks”).

### Strategy coding for the memory tasks

Based on our earlier work on the classification of open-ended memory strategy reports (Fellman et al., 2020; Laine et al., 2018; Waris, Fellman, et al., 2021; Waris, Jylkkä, et al. 2021), as well as expectations concerning PM-related strategies, we devised a coding scheme that is depicted in the online Supplementary Table 1. Two independent raters (M.L. and T.E.) coded each open-ended strategy response. For each participant, there were 10 strategy reports for EPELI (i.e., after each block). The raters coded each strategy report on three variables: the first reported strategy type, the total number of strategy types reported, and the total number of specific strategy details given (be they for one or more strategy types). Interrater agreement for the strategy coding in EPELI was assessed by unweighted kappa (κ) for the first reported strategy type and with linearly weighted kappa (κw) for the other two strategy variables (total number of strategy types reported, total number of specific strategy details). The data for these analyses comprised all participants except for the initial batch of 20 subjects that was used for coding practice. The kappa coefficients for EPELI suggested substantial agreement between the two raters: κ = 0.76 for the first reported strategy type, κw = 0.75 for the total number of strategy types, and κw = 0.73 for total number of specific strategy details. Differences in coding were solved by subsequent consensus meetings.

### Bayesian statistical analysis

All statistical analyses were performed with R version 4.0.0 using the “BayesFactor” package (Morey & Rouder, 2015), and with JASP (version 0.16.3). Our Bayesian statistical analyses applied the default prior setting, that is, Cauchy distribution using a scaling factor *r* = .707. Bayes factor (BF) allows for the assessment of the evidence for the null hypothesis or for the alternative hypothesis on a continuous scale with a range of 1–∞. BF = 1 indicates perfect ambiguity (no evidence for neither hypothesis), whereas a BF above or below 1 indicates evidence for the alternative or null hypothesis, respectively. Interpretation of the BFs followed the guidelines put forth by Kass and Raftery (1995), where BFs between 1 and 3 constitute “weak evidence,” BFs between 3 and 20 as “positive evidence,” BFs between 20 and 150 as “strong evidence,” and BFs > 150 as “very strong evidence.” Together with BFs, we also report the proportional error estimate on the BF approximation (i.e., the uncertainty or imprecision in the BFs), as well as estimates of between-group mean differences using a posterior distribution with 10,000 iterations coupled with their 95% credible intervals.

The relationships between strategy use and the EPELI performance variables were examined with Bayesian Linear mixed effect models (LMEs) with strategy use (dichotomised as yes/no) and block (treated as a continuous variable) as independent variables, and participant as a random factor. This analytical approach made it possible to utilise the whole EPELI data set where the same individual participants could be strategy users and strategy non-users in different task blocks. As the original scales of the EPELI performance variables yield low values, we multiplied them by 1,000 to obtain estimates with a precision of two decimal points. Note that this rescaling does not change the actual results or the inferences that could be drawn from them.

## Results

### Descriptive data on strategy use in EPELI

**Figure 2.**
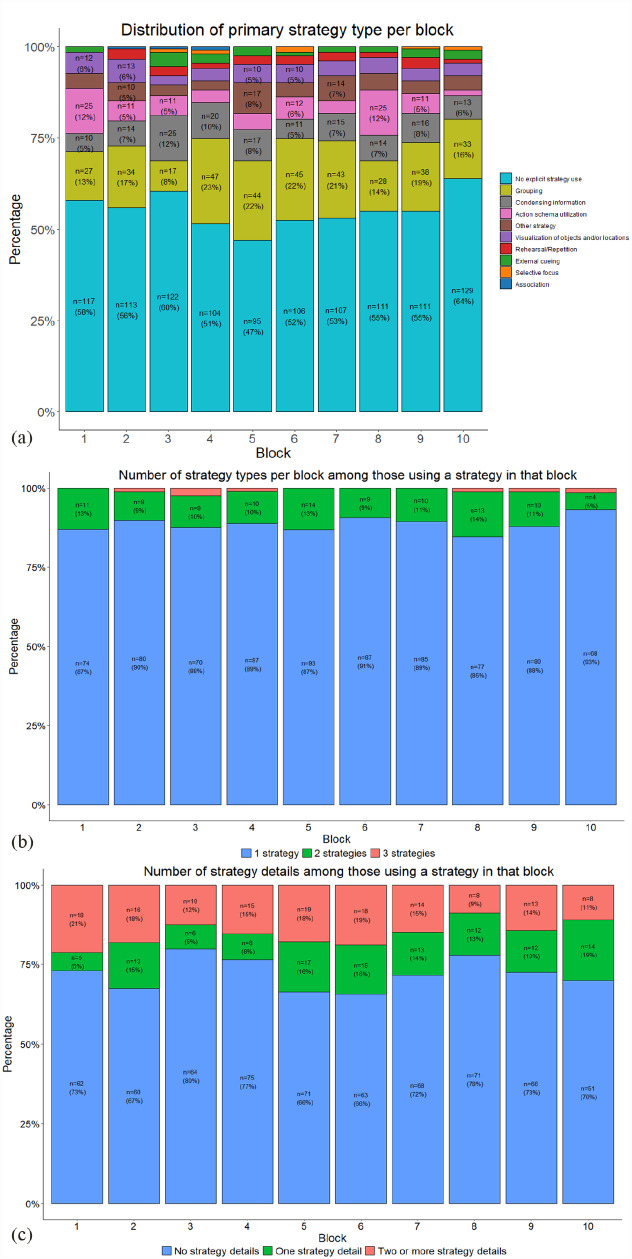
Block-wise distributions of the three strategy-related variables in EPELI. Please see the online version for coloured figures. (a) Distribution of primary strategy type per block. (b) The number of strategy types per block among those using a strategy in that block. (c) Distribution of the reported number of strategy details per block among those using a strategy in that block. For each block, case counts and percentages are provided only for the categories with four or more cases.

Mean performance on the five EPELI main variables is listed in the online Supplementary Table 2. Primary strategy type in the 10 EPELI task blocks is depicted in Figure 2a. It indicates that on average, 45% of the participants reported spontaneous strategy use in the task blocks. The relative proportions of the primary strategy types varied somewhat between the blocks, with the most common ones being grouping (e.g., “I memorized what I had to do in each room”), condensing information (e.g., “Memorized only the crucial contents of the tasks”), and action schema utilisation (e.g., “I do these morning chores every day so it was easy to remember”). Strategy users’ reports revealed most often the employment of a single strategy in a given block, rather than having multiple strategies in use (Figure 2b). With regard to the number of specific strategy details, Figure 2c shows that strategy users’ reports included most often no specific details (e.g., “I did the tasks room by room”).

### Testing the pre-registered hypothesis: strategy use is related to performance in EPELI

**Figure 3.**
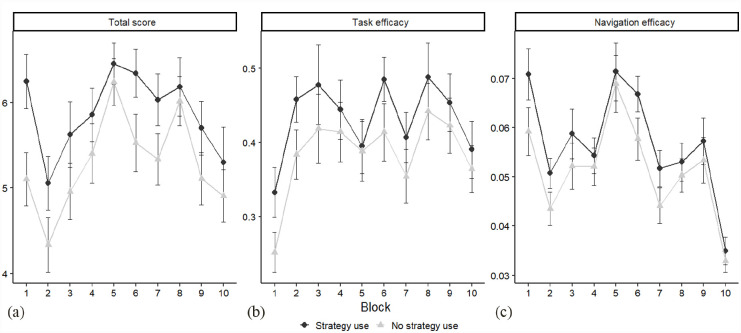
Block-by-block performance in the outcome variables: (a) total score, (b) task efficacy, and (c) navigation efficacy for strategy users versus non-users in a given block (*n* = 202). Error bars represent 95% confidence intervals.

With the *total score* as the outcome variable (Figure 3a), the BF showed very strong evidence of a main effect of strategy (*M*_diff_ = −647.13, 95% highest density interval [HDI] [−717.77 to −576.79], BF_H1_ > 150 ± 1.82%), indicating higher rates of completed subtasks for those using a strategy. Evidence for a main effect of block was weak (*M*_diff_ = 23.43, 95% HDI [−43.66 to 3.42], BF_H1_ = 1.67 ± 2.30%), showing that block-by-block performance as measured by the total score did not change. Finally, there was weak evidence against a strategy × block interaction (*M*_diff_ = 39.33, 95% HDI [19.34–60.90], BF_H1_ = ½.13 ± 2.53%).

With *task efficacy* as the outcome variable (Figure 3b), we observed again very strong evidence for a main effect of strategy (*M*_diff_ = 63.48, 95% HDI [51.74–76.25], BF_H1_ > 150 ± 2.77%), favouring strategy users. Moreover, there was very strong evidence for a main effect of block (*M*_diff_ = 6.64, 95% HDI [2.99–10.58], BF_H1_ > 150 ± 2.24%), reflecting considerable variability between the blocks, with the first block eliciting clearly lowest scores. The evidence for an interaction between strategy and block favoured the null hypothesis with weak evidence (*M*_diff_ = −0.44, 95% HDI [−4.44 to 3.33], BF_H1_ = 1/1.62 ± 2.34%).

As regards *navigation efficacy* (Figure 3c), there was very strong evidence for a main effect of strategy (*M*_diff_ = 9.3, 95% HDI [7.83–10.72], BF_H1_ > 150 ± 2.54%), with strategy users exhibiting higher scores. Very strong evidence for a main effect of block (*M*_diff_ = −1.44, 95% HDI [−1.87 to −0.99], BF_H1_ > 150 ± 5.43%) indicated considerable block-by-block variability. We found positive evidence against strategy use × block interaction (*M*_diff_ = −0.06, 95% HDI [−0.55 to 0.37], BF_H1_ = 1/10 ± 3.44%).

As additional analyses, we performed Bayesian LMEs for elucidating whether a specific primary strategy type or types was associated with the EPELI performance advantages. Because very few participants kept using a single strategy across the task blocks, this analysis was conducted on block-wise use of strategy types in each block, while participant was, as before, treated as a random factor on the intercept. Using pairwise contrasts, “no explicit strategy use” served as the baseline level in the independent variable, whereas a given strategy type served as the other level to which the former one was contrasted. Given that many of the strategies were reported in only few instances, we defined a threshold, stipulating that a given strategy had to be reported in ≥ 10 instances in each block for being eligible for this analysis. Two strategy types reached this threshold, namely, grouping (block with fewest instances *n* = 17) and condensing information (block with fewest instances *n* = 10).

**Figure 4.**
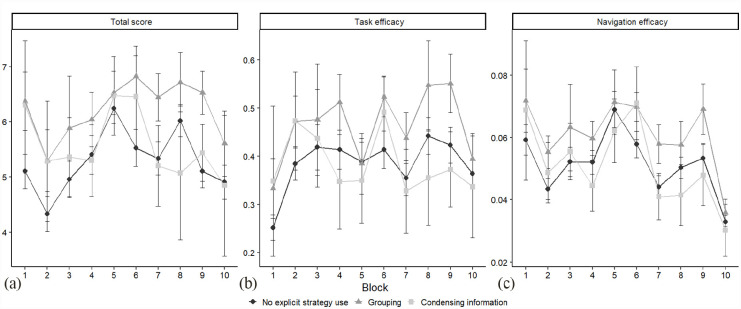
Block-by-block performance on the outcome variables: (a) total score, (b) task efficacy, and (c) navigation efficacy for strategy non-users, grouping strategy users, and those using the strategy of condensing information. Note that the constellations of participants vary between the blocks, as a given participant can belong to different strategy categories in different blocks. Error bars represent 95% confidence intervals.

The results depicted in Figure 4 showed very strong evidence for grouping strategy, as compared with no strategy use, being advantageous concerning total score (*M*_diff_ = 786.47, 95% HDI [690.03–887.79], BF_H1_ > 150 ± 2.83%), task efficacy (*M*_diff_ = 63.48, 95% HDI [51.74–76.25], BF_H1_ > 150 ± 4.67%), and navigation efficacy (*M*_diff_ = 9.30, 95% HDI [7.83–10.72], BF_H1_ > 150 ± 1.91%). This was not the case for condensing information; the results here showed moderate evidence against an effect of strategy type on each EPELI performance measure total score (*M*_diff_ = 229.38, 95% HDI [66.29–380.49], BF_H1_ = 1/3.03 ± 2.18%; task efficacy (*M*_diff_ = −5.11, 95% HDI [−23.79 – 13.07], BF_H1_ = 1/11.11 ± 5.88%; navigation efficacy (*M*_diff_ = −0.37, 95% HDI [−2.41 to 1.87], BF_H1_ = 1/11.11 ± 4.53%). Evidence against a strategy type × block interaction was either weak or positive for both grouping and condensing information on each of the three dependent variables (BF_H1_ range: 1/10.00–1/1.15). All in all, our results indicate that grouping, but not condensing information, was linked to higher EPELI performance as compared with no strategy use (Figure 4).

### Research question: does strategy use evolve during the 10 EPELI task blocks?

**Figure 5.**
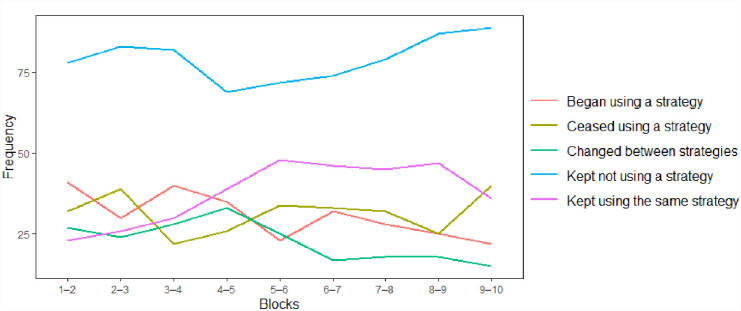
Changes in strategy use over the 10 EPELI task blocks. Number of observations (individuals) shown for each of the nine block transitions.

Figure 2 does not reveal any evident trends in the primary types of strategies employed over the 10 blocks. However, as shown in Figure 5, block transitions in strategy use indicate that the number of participants relying on the same strategy as in a previous block doubled during the first five block transitions. There were concomitant downwards trends in strategy pickers (going from no strategy to some strategy) and strategy changers (going from one active strategy to another active strategy) over the block transitions. Overall, this descriptive pattern suggests a gradual stabilisation in strategy use as the participants were becoming more familiar with EPELI. At the same time, over the whole task period a substantial proportion of participants remained strategy non-users when moving from one block to another.

### Research question: is there congruity in participants’ strategy use over the three memory tasks?

The strategy use intercorrelations with the corresponding BFs are reported in Table 1. The results yielded positive evidence for an association in strategy use between EPELI and Word List Learning (*r* = .212, BF_10_ = 3.97), so that more frequent use of a strategy in EPELI is related to more common strategy employment in Word List Learning as well. However, the proportion of shared variance is quite low (4.5%). Strategy use in the EPELI Instruction Recall task was not associated with strategy use in the other two memory tasks, but it should be noted that the task may have been less sensitive as it consisted of only a single block.

**Table 1.** Intercorrelations (Pearson’s *r*) and corresponding Bayes factors (BF_10_) of strategy use in EPELI, EPELI Instruction Recall, and Word List Learning.

Variable	EPELI	EPELI Instruction Recall
EPELI Instruction		
Recall	*r* = .178	
	BF_10_ = 1.45	
	*n* = 173	
Word List Learning	*r* = .212	*r* = .067
	BF_10_ = 3.97	BF_10_ = 0.14
	*n* = 166	*n* = 166

EPELI: Executive Performance in Everyday LIving; BF: Bayes factor.

Strategy use defined as the number of task blocks where a participant reported employing a strategy.

## Discussion

The aim of this study was to examine spontaneous use of internal strategies in adults when they were performing common daily chores from memory in a virtual setting. The analyses were expected to shed light on the employment of self-generated memory strategies in life-like situations. More specifically, we tested the hypothesis that spontaneous strategy use is associated with better performance in the EPELI game. Moreover, we explored the possible development of strategy use over the 10 task blocks, and congruity in strategy use over three different memory tasks. The results provided clear support for our pre-registered hypothesis, as strategy use compared with non-use was associated with better EPELI performance. With regard to the two additional research questions, strategy use appeared to evolve gradually over the task blocks as strategy changes became less common, and we saw some (rather weak) congruity in the tendency to employ a strategy in EPELI and in an episodic memory task, Word List Learning.

The present very strong evidence (BF_H1_ > 150) for a better outcome on the three EPELI performance measures for strategy users than non-users is in line with similar results obtained from the episodic memory and working memory domains (Dunlosky & Kane, 2007; Dunning & Holmes, 2014; Fellman et al., 2020; Forsberg et al., 2020; Laine et al., 2018; Malinovitch et al., 2021; McNamara & Scott, 2001; Turley-Ames & Whitfield, 2003; Waris, Fellman, et al., 2021; Waris, Jylkkä, et al. 2021), as well as laboratory PM tasks (Reese-Melancon et al., 2019). Indeed, both episodic memory and working memory, as well as executive functions, are considered to be central cognitive components underlying PM (McDaniel & Einstein, 2011; Zuber & Kliegel, 2020), and those components are certainly involved in our complex task as well. The present results serve to expand the findings on the positive link between strategy use and memory task performance, as EPELI in certain respects more closely mimics real-life conditions compared with classical experimental PM tasks. Also in contrast to classical PM tasks, it represents a mixture of “shopping list”-type tasks, time-based tasks, and an event-based task, with the first category being by far the most common task type. Moreover, implementation of intentions followed right after the instructions and there was no ongoing non-PM task. Given our setup, one could assume that part of the EPELI instructions remained in working memory, but as each block included seven to eight different tasks, EPELI is likely a supra-span memory task (i.e., partially dependent on episodic memory). The engagement of both working memory and episodic memory in EPELI does not necessarily affect the present strategy-related findings, as both memory systems should benefit from very similar strategies that either help to strengthen the memory trace (e.g., rehearsal/repetition), decrease the memory load by clustering the to-be-remembered actions into fewer units (grouping, condensing information), or link them to existing relevant long-term memory representations (e.g., action schema utilisation).

Regarding specific primary strategies, their varying block-wise distributions and insufficient rates of observations limited the exploration of possible performance differences to three strategy types, namely, no strategy (serving as baseline), grouping and condensing information. We obtained very strong evidence (BF_H1_ > 150) for users of grouping strategy, but not those with condensing information, performing better on the three EPELI measures than strategy non-users. Thus, these results highlight one particular strategy that appears to be well applicable and clearly useful in implementing intentions in the complex EPELI contexts. It is also interesting to note that besides grouping, the most common primary strategy types were condensing information and action schema utilisation.

These strategies differ markedly from the by far most common strategies (monitoring/maintenance rehearsal, keeping up a sensory-motor readiness to perform the PM task) reported by Reese-Melancon et al. (2019) in a laboratory PM task. This discrepancy prompted us to examine the participants’ use of rehearsal/repetition in EPELI Instruction Recall: its proportion turned out to be 15.8%, higher than the corresponding average rate in EPELI (2%). We assume that the open-endedness of EPELI, together with the richer context and semantically meaningful block-wise scenarios as compared with common PM laboratory tasks, trigger the use of strategies that involve manipulation of to-be-remembered information, rather than monitoring/maintenance rehearsal. In other words, task characteristics play a considerable role in the repertoire of self-generated strategies that a given task elicits (see also Morrison et al., 2016). The fundamentally different task characteristics between EPELI and the common PM laboratory tasks are the likely reason for the fact that in contrast to the laboratory tasks, active monitoring for environmental cues (the external cueing strategy) appeared as a primary strategy in only 0%–4% of our strategy reports. Perhaps in most cases participants first performed all the EPELI tasks they had in memory following whatever strategy they had chosen (and report that as their primary strategy), and resorted to intentional use of external cueing only afterwards if there was still some time left to finish off the task block. The richness of EPELI’s virtual surroundings may also decrease the salience of individual environmental cues.

The interest in probing possible strategy evolvement over the single-session testing with EPELI stems from recent empirical evidence that indicates fast changes in strategy use in experimental working memory and episodic memory tasks (Waris, Fellman, et al., 2021; Waris, Jylkkä, et al. 2021). More specifically, these two studies found that increases in strategy use and changes in self-reported strategy were most common in the initial task block transitions. These findings were interpreted in the context of the general cognitive skill learning framework (e.g., Chein & Schneider, 2012): with complex tasks, the metacognitive system is initially engaged in generating strategies to handle the task. The block-wise findings from EPELI did not show any systematic changes in the proportion of participants employing a strategy, but block-by-block transitions suggested a similar decreasing trend in strategy changers as Waris, Fellman, et al. (2021); Waris, Jylkkä, et al. (2021) found in their studies. This hints at a gradual stabilisation in strategy employment within the single EPELI test session, suggesting that this pattern holds also for a complex everyday memory task. Gradual changes in self-reported PM strategy use, with overall increase in strategy use and more frequent employment of strategies deemed as more effective, have been reported earlier, but the time span was very different, a 4-week training period with an adaptive PM task (Rose et al., 2015).

Possible convergence in strategy use between different memory tasks relates to the more general issue of determinants of strategy employment. In case individuals vary in their overall degree of strategy proneness, strategy use across tasks should show a positive correlation. We found some evidence for this in the form of a positive association in strategy use between EPELI and Word List Learning, an experimental episodic-verbal memory task. However, this association was rather weak, which could suggest that the decision to use vs. not to use a strategy reflects a complex interplay of task features and participant characteristics. Potentially relevant characteristics in this respect include general cognitive ability (Pizzonia & Suhr, 2022) and metacognitive features such as one’s self-efficacy and control beliefs (Hutchens et al., 2013). Finally, one could note that the lack of correlations with the EPELI Instruction Recall task may have been due to the minimal range (0 or 1) of the variable in question.

### Limitations

There are certain limitations to the present study that should be considered. First, as strategy data stems from verbal self-reports to a general open-ended question, we cannot be sure that all strategies employed were reported. There is previous evidence suggesting that open-ended questions may overestimate the proportion of no strategy users that was quite high (on average 55%) in the present study. Waris, Jylkkä, et al. (2021; see Table 8) administered both an open-ended strategy question and a list-based strategy question after the last block of an adaptive working memory task. The open-ended question yielded 32% of no strategy users, while for the immediately following list-based strategy report that concerned the same task block, only 7% of the same participants chose the “no strategy” alternative. Nevertheless, the fact that reported strategy use was strongly related to EPELI performance supports the conclusion that open-ended strategy reports can catch a significant part of actual strategy use. EPELI raw data including each mouse click and movement could give an opportunity to objectively verify some reported strategy types, such as executing the tasks in a certain room order. We are currently looking into this in another study. Second, as the block-wise strategy reports were retrospective, planning and execution stages cannot be separated here (see Kliegel et al., 2000). For future studies, it would be possible to insert a planning stage with an explicit report between the task instructions and actual EPELI performance. Third, as noted above, comparisons with studies employing classical PM tasks are complicated by the fact that the current EPELI version differs from them by consisting mostly of shopping list type tasks. Although this makes the comparison between EPELI and more traditional time-based or event-based PM less straightforward, we would argue that various to-do-lists with multiple steps of action are relevant for PM research, as they are not uncommon in everyday life. In contrast to classical PM tasks, performance in EPELI is initiated right after the instructions and there is no separate ongoing task, but it seems likely that moving, exploring and acting upon various objects in a rich environment makes EPELI different from mere list recall (cf. also the remarkably low rates of maintenance strategy use in EPELI discussed above). Of course, task lists or blocks represent only one aspect of everyday memory challenges that can appear in different task contexts and undergo shifts in their priorities. Fourth, as we focussed on internal strategies, the use of external memory aids was prohibited in the task instructions and participants reporting the use of such aids were excluded from the analyses. Nevertheless, it can be argued that this affects the external validity of our study, as the employment of various external memory aids is part and parcel of everyday life (e.g., Harris, 1980), and the alternatives have increased with today’s digital reminders and calendars. On the other hand, our focus on internal strategies made the results more comparable with similar earlier studies, revealing interesting differences in most commonly employed spontaneous internal strategies. Fifth, as this was an online study, we had not control over the conditions under which the participants performed the tasks, albeit the instructions emphasised the need for a quiet space. Nevertheless, the exclusion criteria and removal of outliers should have helped to counteract this potential problem. Moreover, research indicates that online testing successfully replicates the cognitive task effects observed in the lab (e.g., Enochson & Culbertson, 2015; Germine et al., 2012; Waris et al., 2017). Also for PM tasks, there is evidence for a comparable performance at laboratory and at home (Zuber et al., 2022). Sixth, it is important to point out that the present evidence for the link between strategy use and EPELI performance is correlative. However, numerous intervention studies on PM strategy training and its effect on task performance have indicated a causal relationship between strategy use and PM performance (for a meta-analysis, see Jones et al., 2021).

### Implications

What are the implications of the present results concerning everyday memory performances? It seems evident that despite EPELIs apparent verisimilitude, the present simulated everyday task lists given in a testing context differ from daily life. In the latter case, the surroundings are most often very familiar and richer and more dynamic, memory tasks are more varied in terms of contents, habituality, priority, time span, and environmental cues, both external and internal strategies are in use, and goals are most often set by the individual. Moreover, in everyday life memory performance does not take place in a testing situation which may bring up maximal rather than typical performance (e.g., Toplak et al., 2013). Instead, we would argue that the present results give insight into the potential for everyday internal memory strategy use in adults by revealing the variety of strategy types—the most common ones here being grouping, action schema utilisation, condensing information—that they can generate by themselves when faced with life-like tasks that load on memory and executive performance. In the present task, grouping turned out to be a particularly effective strategy, and it can be readily applied in everyday life (consider, e.g., shopping for groceries that could be helped by organising the shopping list into subcategories such as dairy products, fruits and vegetables, bakery that are located in separate sections in a supermarket). Goal-oriented grouping or chunking is considered to be a fundamental cognitive mechanism that facilitates learning and memory (Gobet et al., 2001). Moreover, it has been shown that a related concept, meaningful event segmentation, is related not only to better real-life memory (McGatlin et al., 2018) but also to better action performance (Bailey et al., 2013). Thus, information on grouping and other naturally occurring strategies as well as their efficacy should also be helpful when planning further memory interventions that incorporate strategy training.

## Supplemental Material

sj-docx-1-qjp-10.1177_17470218231183958 – Supplemental material for Spontaneous memory strategies in a videogame simulating everyday memory tasks Supplemental material, sj-docx-1-qjp-10.1177_17470218231183958 for Spontaneous memory strategies in a videogame simulating everyday memory tasks by Matti Laine, Jussi Jylkkä, Liisa Ritakallio, Tilda Eräste, Suvi Kangas, Alexandra Hering, Sascha Zuber, Matthias Kliegel, Daniel Fellman and Juha Salmi in Quarterly Journal of Experimental Psychology

## Notes

1 Jones et al. (2021) employed the following criteria for ecological validity ratings of PM assessments: did the assessment employ a delayed-intention task rather than merely a self-report, did it take place outside the laboratory setting, did one include participant-generated tasks based on participants’ personal daily activities.
