# Supplementary material for: Spontaneous memory strategies in a videogame simulating everyday memory tasks
Source: Q J Exp Psychol (Hove). 2023 Jul 3;77(3):611–25. doi: 10.1177/17470218231183958 (PMC10958750; doi:10.1177/17470218231183958)
Supplement: sj-docx-1-qjp-10.1177_17470218231183958 – Supplemental material for Spontaneous memory strategies in a videogame simulating everyday memory tasks [file sj-docx-1-qjp-10.1177_17470218231183958.docx]

Supplementary Material for:

**SPONTANEOUS MEMORY STRATEGIES IN A VIDEOGAME SIMULATING EVERYDAY MEMORY TASKS**

Matti Laine^1^, Jussi Jylkkä^1^, Liisa Ritakallio^1^, Tilda Eräste^1^, Suvi Kangas^2^, Alexandra Hering^3,4^, Sascha Zuber^5,6^, Matthias Kliegel^4,5^, Daniel Fellman^1^, & Juha Salmi^2,7^

^1^Department of Psychology, Åbo Akademi University, Finland

^2^Department of Psychology, University of Helsinki, Finland

^3^Department of Developmental Psychology, Tilburg University, The Netherlands

^4^Faculty of Psychology and Educational Sciences, University of Geneva, Switzerland

^5^Centre for the Interdisciplinary Study of Gerontology and Vulnerability, University of Geneva, Switzerland

^6^Swiss National Center of Competences in Research LIVES—Overcoming Vulnerability: Life Course Perspectives, Lausanne & Geneva, Switzerland

^7^Department of Neuroscience and Biomedical Engineering, Aalto

University, Finland

Corresponding author: Matti Laine, Department of Psychology, Åbo Akademi University, Fabriksgatran 2, 20500 Turku, Finland. Email matti.laine@abo.fi

**Supplementary 1. The memory strategy coding scheme**

**GENERAL INSTRUCTIONS FOR CODING PARTICIPANTS’ OPEN-ENDED BLOCK-WISE MEMORY STRATEGY DESCRIPTIONS IN THE ADULT EPELI EXPERIMENT**

- For each strategy description, code three variables: the first reported strategy type (there can be only one primary strategy), the total number of strategy types reported, and the total number of specific strategy details given (be they for one or more strategy types).

o If the strategy description field is completely empty, do not code anything.

o If the participant has typed in “None” but nevertheless given a subsequent description of a strategy, evaluate the response from the strategy description.

o If the participant refers to a strategy s/he used in a previous task block, evaluate the response from the strategy description for that other block.

o If the participant refers to a strategy s/he used for another block that cannot be identified on the basis of the response, mark this as “other strategy”.

o Extraneous information not related to strategy use such as “it went bad”, “the task was difficult”, “my mom disturbed me during task performance” should not be taken into account when rating the strategy descriptions, neither comments that merely describe the sequence of actions one took when performing the task block, or comments that merely recount the task characteristics or the task instructions. Keep in mind that strategy is an identifiable, self-generated way or ways of managing a memory task, thus requiring activity from the part of the participant.

o Possible use of external aids (e.g., “wrote down what needed to be done”) are coded as 999, as these participants will be discarded from the strategy analyses

o The same coding schema for strategy type and strategy level of detail is used for all three tasks where strategy report is collected, namely the EPELI task (10 blocks, strategy report for each block), the EPELI Instruction Recall task (1 block), and the Word List Learning task (3 blocks). Note however that not all strategy types are used in coding all these three tasks.

| **Code for strategy type** | **Type** | **Examples for EPELI** | **Examples for Word List Learning** |
| --- | --- | --- | --- |
| **0** | **No explicit strategy use**   - No strategy - No clear evidence of strategy use - Repetition of instructions or own action sequence - Response unrelated to strategy use, comments on understanding instructions, or comments on task difficulty - Guessing, intuition, instinct, familiarity, ‘gut feeling’ | - “No”, “None” - “Most of the tasks I remembered, had actually only trouble in remembering to drink the glass of milk” - “At first I made the bed, then put on clothes, drank coffee, ate sandwich and finally moved to the study to turn on the lights but the time ran out.” - “This one was hard, I got distracted” - “Followed the same order as was given in the instructions” - “Just guessed and clicked on many things” - “Used my memory” - “By trial and error” - “I listened very carefully” - Only “Yes” or “I used a strategy” or “Attempted to use a mnemonic” (with no description as to what the strategy/mnemonics would have been) | - “No system” - “I tried” - “I tried to keep up with the list but found it quite difficult” - “Just tried to concentrate on the words” - “I memorized the words mentally” - “I memorized all the words” - “Tried to apply a strategy/mnemonic”(with no description as to what the strategy/mnemonics would have been) |
| **1** | **Rehearsal / Repetition**   - Rehearsing items verbally, nonverbally or visuomotorically - *E.g. repeating, speaking in head, saying in mind, yelling the tasks, rehearsing quietly* | - “Memorized the instructions by going through them silently in my mind” - “Rehearsed the tasks in my mind” - ”Said out aloud all the instructions” | - “I repeated the words” - “I rehearsed the words in my mind” - “Said aloud all the items in the list in order to keep them in memory” |
| **2** | **Grouping**   - Grouping items together - *E.g. chunking, pairing, memorizing in groups, remembering in chunks, thinking in pairs, dividing into subsets, subdividing into sets, splitting into shorter sequences* | - “I memorized what I had to do in each room, starting with the bedroom” - “Remembered the tasks room by room” - “Tried to group tasks by their location” - “Grouping tasks by their type, like handling foodstuff or cleaning-related chores” | - “I memorized the words in chunks” - “I grouped the words in pairs” - “I remembered the words in groups” - “grouped together in my mind words that rhymed” |
| **3** | **Action schema utilization**   - *E.g. thinking what one would do at home, thinking of normal household chores, reflecting on what one would do in a similar scenario* | - “I tried to think what I usually do before leaving for work” - “These made much sense as they reminded me of things I would do when cleaning the house” - “I do these morning chores every day so it was easy to remember” - “Applied logic based on my own routines” - “Followed my standard routine” | - N/A |
| **4** | **Association**   - Associating the objects/actions with something outside the stimuli | - “Associated each task with a letter and then memorized that letter sequence” - “Thought of different colors to which I associated the tasks” | - “I tried to associate each word with something that I have at home” |
| **5** | **Visualization of objects and/or locations**   - Linking tasks with some visual/visuospatial representations in mind - *E.g. visualizing objects in mind, forming spatial images, visualizing objects in their locations* | - “I tried to see the relevant objects in my mind” - “Attempted to visualize the objects and what I was supposed to do with them” - “Tried to see myself going through the apartment and doing the things I was supposed to do” - “Imagined in my mind how I would do the tasks” - “Pictured the tasks in my head” | - “Built a mental image of the words in my mind” - “Visualized the words as they were shown” - “I was picturing the words in my head” |
| **6** | **Condensing information**   - Abridging the instructions e.g. into keywords | - “I created just one key word for each task” - “Memorized only the crucial contents of the tasks, not the whole sentence” | - N/A |
| **7** | **Selective focus**   - Focusing on only some of the tasks or keywords for the tasks | - “I focused on remembering only a subset of the items and disregarded the rest” - “This was difficult, I just concentrated on the easy tasks” - “Fixated only on the first 4 tasks” | - “I focused on remembering only a subset of the words and disregarded the rest” - “Just concentrated on the easy words as the task was quite difficult” - “Focused on the words I had previously forgotten” - “Fixated only on the first 4 words” |
| **8** | **External cueing**   - Using objects in the apartment as memory/action triggers | - “My memory failed so I resorted to using the things in the apartment as prompts” - “Walked around and looked at various things that reminded me of the tasks I had to do” - “Some visual clues like seeing the teddy bear helped to trigger my memory” - “Looked for visual reminders in the rooms” | - N/A |
| **9** | **Other strategy** | - “I put the actions I was supposed to do in a storyline” - “Made up a song based on the instructions” - “Employed an elimination strategy” - “Started with the task that was most difficult to remember” - “Made a list of hierarchies in my mind” | - “Made up a story based on the words to remember them better” - “I tried to match the words to those I saw previously” |

| **Code for strategy level of detail** | **Level** | **Examples for EPELI** | **Examples for Word List Learning** |
| --- | --- | --- | --- |
| **0** | **No strategy use**   - No strategy use - Vague comments that do not point to an identifiable strategy | - “No system” - “I tried” - “I tried to keep up but found it quite difficult” - “Just tried to concentrate on the instructions” - “I memorized the tasks mentally” - “I memorized all the instructions” - “Tried to apply a strategy/mnemonic” | - “No system” - “I tried” - “I tried to keep up but found it quite difficult” - “Just tried to concentrate on the words” - “I memorized the words mentally” - “I memorized all the words” - “Tried to apply a strategy/mnemonic” |
| **0** | **An identifiable strategy or strategies without any specific detail** | - “Grouped the tasks to help to remember them” *(grouping)* - “I rehearsed the tasks” *(rehearsal)* - “I repeated the instructions as they came, and then divided them into groups” *(rehearsal, grouping)* | - “Grouped the words to help to remember them” *(grouping)* - “I rehearsed the words” *(rehearsal)* - “I repeated the words as they came, and then divided them into groups” *(rehearsal, grouping)* |
| **1** | **An identifiable strategy or strategies with one specific feature in total** | - “I tried to remember the tasks in groups of 3 actions” *(grouping/groups of 3)* - “Related the tasks to my own morning routines like washing my face after waking up” (*internal cueing/face washing*) - “Did room by room, starting from the nearest room” *(grouping/start room)* - “I repeated the tasks silently in my mind and then memorized by grouping them room by room like doing the kitchen tasks first (*rehearsal*, *grouping/kitchen*) | - “I tried to remember the words in groups of 3” (grouping/groups of 3) - “I repeated the words silently in my mind and then memorized by associating them with something, like apple – eating a delicious apple pie (*rehearsal, association/apple*) |
| **2** | **An identifiable strategy or strategies with two specific features in total** | - “I grouped the tasks room by room and started from the bedroom then the kitchen” *(grouping/bedroom, kitchen)* - “I visualized the tasks like seeing myself driving the exercise bike and used also keywords like ‘wash’ to remember *(visualization/driving exercise bike, keywords/wash)* | - “I created images in my mind, like a cat on the sofa and a king in a castle (*association/2 examples*) - “Focused on the words I forgot on the last round and made links between them, like the cat hopping on the king and the eagle landing on a mountain” (*selective focus, association/2 examples*) |
| **3** | **An identifiable strategy or strategies with at least three specific features in total** | - “I associated the tasks with each other, like tooth-brushing – hand washing, placing a book and a magazine in the right place, eating a sandwich while having a cup of tea” (*association/3 specific examples*) - “This was a difficult one -- I focused on only the four last tasks and grouped them by the rooms: things to do in the kitchen and the living room” (*selective focus/four last tasks*, *grouping/2 specific examples*) | - I linked the words with familiar places, like chair – my favourite armchair in our livingroom, mountain – Mt Snowdon in Wales, castle – the Windsor castle (*association/ 3 examples*) |
| **4-** | **…and so on** |  |  |

| **Supplementary Table 2. Number and percentage of strategy use** | | | | | | | | | | |
| --- | --- | --- | --- | --- | --- | --- | --- | --- | --- | --- |
|  | **Block** | | | | | | | | | |
| **Strategy type** | 1 | 2 | 3 | 4 | 5 | 6 | 7 | 8 | 9 | 10 |
| Action schema utilization | 25 (12.4%) | 11 (5.4%) | 11 (5.4%) | 7 (3.5%) | 9 (4.5%) | 12 (5.9%) | 7 (3.5%) | 25 (12.4%) | 11 (5.4%) | 3 (1.5%) |
| Association | 0 (0%) | 1 (0.5%) | 1 (0.5%) | 2 (1%) | 0 (0%) | 0 (0%) | 0 (0%) | 0 (0%) | 0 (0%) | 0 (0%) |
| Condensing information | 10 (5%) | 14 (6.9%) | 25 (12.4%) | 20 (9.9%) | 17 (8.4%) | 11 (5.4%) | 15 (7.4%) | 14 (6.9%) | 16 (7.9%) | 13 (6.4%) |
| External cueing | 3 (1.5%) | 0 (0%) | 8 (4%) | 5 (2.5%) | 5 (2.5%) | 2 (1%) | 3 (1.5%) | 3 (1.5%) | 5 (2.5%) | 5 (2.5%) |
| Grouping | 27 (13.4%) | 34 (16.8%) | 17 (8.4%) | 47 (23.3%) | 44 (21.8%) | 45 (22.3%) | 43 (21.3%) | 28 (13.9%) | 38 (18.8%) | 33 (16.3%) |
| No explicit strategy use | 117 (57.9%) | 113 (55.9%) | 122 (60.4%) | 104 (51.5%) | 95 (47%) | 106 (52.5%) | 107 (53%) | 111 (55%) | 111 (55%) | 129 (63.9%) |
| Other strategy | 8 (4%) | 10 (5%) | 6 (3%) | 5 (2.5%) | 17 (8.4%) | 8 (4%) | 14 (6.9%) | 9 (4.5%) | 7 (3.5%) | 8 (4%) |
| Rehearsal/Repetition | 0 (0%) | 6 (3%) | 5 (2.5%) | 3 (1.5%) | 5 (2.5%) | 5 (2.5%) | 5 (2.5%) | 3 (1.5%) | 6 (3%) | 2 (1%) |
| Selective focus | 0 (0%) | 0 (0%) | 2 (1%) | 2 (1%) | 0 (0%) | 3 (1.5%) | 0 (0%) | 0 (0%) | 1 (0.5%) | 2 (1%) |
| Visualization of objects and/or locations | 12 (5.9%) | 13 (6.4%) | 5 (2.5%) | 7 (3.5%) | 10 (5%) | 10 (5%) | 8 (4%) | 9 (4.5%) | 7 (3.5%) | 7 (3.5%) |
| Note. Numbers outside from parentheses shows actual n, and numbers within parentheses the percentage of the total in a given block. N = 202 | | | | |  |  |  |  |  |  |
